# Striving Toward Equity in Health Care for People With Communication Disabilities

**DOI:** 10.1044/2022_JSLHR-22-00057

**Published:** 2022-07-18

**Authors:** Megan A. Morris

**Affiliations:** a Division of General Internal Medicine, Department of Medicine, University of Colorado Anschutz Medical Campus, Aurora

## Abstract

**Purpose:**

Approximately 10% of the U.S. adult population has a speech, language, and/or voice disability, collectively referred to as communication disabilities. An increasing number of studies demonstrate that persons with communication disabilities have worse health and health care outcomes as compared to those without communication disabilities. Understanding the state of the science, including potential contributing factors is critical to begin to address the disparities.

**Method:**

Applying a historical lens and integrating multiple models of disability provide a comprehensive perspective of the health and health care outcomes of persons with communication disabilities.

**Results:**

Three phases for addressing health care disparities exist: detecting, understanding, and reducing. Results from a 2012 National Health Interview Survey provide compelling population-level results of the health and health care disparities experienced by persons with communication disabilities. To understand the disparities, factors within the health care system, such as availability of communication aids and services, as well as provider and staff biases, assumptions, and lack of knowledge need to be considered. To date, few interventions exist to address disparities in care for persons with communication disabilities. Consequently, researchers need to engage with stakeholders in innovative study designs and methods to facilitate the rapid development, implementation, and dissemination of interventions that address the disparities.

**Conclusion:**

To ensure equity for the large and growing population of persons with communication disabilities, researchers, policy makers, patients, and health care systems need to collaborate in identifying and addressing the factors contributing to health and health care disparities.

**Presentation Video:**

https://doi.org/10.23641/asha.21215804

As clinicians, we all have clients who are engrained in our memories. For me, one of those cases is Steven Lee (pseudonym). Steven was a 4-year-old boy with severe apraxia of speech of unknown origin. He was enrolled in his local school's early intervention preschool. As the school's augmentative and alternative communication (AAC) specialist, I was asked to evaluate Steven to determine if he would benefit from a speech-generating device. During my evaluation, Steven excelled at using the device, thrilled with finally having a way to effectively communicate with peers and teachers. He went from not participating in circle time to requiring instruction that he needed to wait for his turn to “speak.” I quickly wrote the evaluation and submitted a request to his private health insurance company to cover the speech-generating device. Weeks later, we received a denial. We submitted additional evidence for why Steven needed the device, including citing evidence that AAC users experience high rates of abuse and so having a means to communicate is critical (Nelson Bryen et al., 2003). Despite this, the insurance company denied coverage of the device a second time. The stated rationale for denial was that, as a 4-year-old, Stephen had his family who could communicate for him, and therefore, he did not need a means to communicate auditorily. Furthermore, they stated that children who are abused typically do not talk about their abuse and, thus, it did not serve as a valid justification for why Steven should have a mechanism to auditorily communicate. In the meantime, while we were advocating to the insurance company, Steven cut his foot. We never knew how or when he cut his foot until he became ill and required emergency care because the cut became infected.

Fortunately, this case had a positive ending. The school district interceded and paid for the speech-generating device for Steven, and he went on to become a proficient AAC user and successful student. Additionally, his foot healed with no further complications. This example demonstrates multiple misconceptions and biases about persons with communication disabilities. Negative biases contributed to Stephen's avoidable poor health care outcome: a costly emergency care visit for an infected cut. Unfortunately, Steven is not a unique case. A growing body of evidence demonstrates that persons with communication disabilities experience significant health and health care disparities, which are due in part to misconceptions and biases. The following review article is a description of the documented disparities. I will begin by describing the models of disability, the disparities experienced by patients with communication disabilities, factors that contribute to these disparities, and then steps as well as potential innovations to mitigate the factors that affect the health and health care outcomes of persons with communication disabilities.

## Models of Disability

The concept of disability has a long and complicated history in our country, the effects of which still echo throughout society today. For example, until the mid-20th century, institutionalization of persons with disabilities was both accepted and expected of families (Shapiro, 1994). As the U.S. population grew, so did the population of persons with disabilities living in institutions. With swollen populations that grew beyond institutions' capacities, abuse and poor living conditions became rampant (Shapiro, 1994). As the public became aware of the atrocities occurring within institutions, in part due to the Willowbrook documentary in 1972, a movement began to deinstitutionalize persons with disabilities, moving them to community settings (Shapiro, 1994). At the time of deinstitutionalization, one of the “solutions” was to move persons with disabilities into nursing homes, contributing to the growth of the nursing home industry (Yohanna, 2013). This legacy lives on today and continues to impact the lives and health of persons with disabilities. In 2020 at the beginning of the COVID-19 pandemic, the implications of such large numbers of people, including those with disabilities living in nursing homes, became very apparent. COVID-19 spread quickly in congregate living settings like nursing homes, leading to high prevalence of COVID-19 infection and death among nursing home residents (Ouslander & Grabowski, 2020). As a result, there are renewed calls for more integration of persons with disabilities into community settings. Environmental factors driven by a history of bias continues to affect the lives and health of people with disabilities today.

Another example of society's problematic past treatment of persons with disabilities is the eugenics movement, which began in the late 19th century in the United States. A core tenet of the eugenics movement was that a person's “bad” qualities are the result of “bad” genes. Bad qualities could include social positions, such as poverty, but also include physical, cognitive, or mental disabilities. Because genes can be inherited, persons with disabilities were sometimes forcibly sterilized in order to prevent introducing more “defective” people into society (Reilly, 2015). Additionally, families were praised if none of their members were “defective,” demonstrating that the family carried good genes. Again, this perpetuated the institutionalization of persons with disability in order to “hide” them from society. While the final forced sterilization laws were stricken from the books in the mid-1980s, we still see traces of the same beliefs today (Stern et al., 2017). This can be summarized in the medical model of disability. Just as the eugenics model argues that impairments are located within an individual, the medical model of disability postulates that disability results from an impairment within the individual (Roush & Sharby, 2011). To mitigate the effects of the disability, the focus is on “fixing” or minimizing the impairment. The medical model of disability can be seen both within the fields of rehabilitation, which often focuses on minimizing or eliminating the impairment, and public health, which aims to prevent impairments and thus disabilities. While no one would argue that impairments do not exist and are not a core component of disability, the medical model perhaps does not offer a comprehensive way to address disability.

The social model of disability, which rose in popularity and acceptance with the disability civil rights movement in the 1970s, states that disability is the result of environmental and societal limitations, restrictions, biases, and assumptions (Roush & Sharby, 2011; Shapiro, 1994). The social model moves disability from existing within the individual to existing within many layers of society, calling for accessibility and inclusion. Instead of “fixing” the individual, society needs to be “fixed.” The classic example of the social model is demonstrated by the following: A person who uses a wheelchair is not disabled until they reach a building with no ramps or elevators. If the building is fully accessible, then the person in the wheelchair would not experience a disability. Therefore, the environment, in this case the building, needs to be “fixed,” rather than the individual. While the social model of disability perhaps is an extreme view of disability, it played an important role in the civil rights movement in shifting our culture's beliefs about disability and continues to have significant implications today, especially as we aim to address disparities in health and health care outcomes.

A perhaps more comprehensive view of disability integrates both the medical and social models of disability. The World Health Organization's International Classification of Functioning, Disability and Health (ICF) exemplifies this integration. In this model, disability is said to result from a complex interaction of a health condition, body function and structure, activities, participation, and contextual factors (World Health Organization, 2003). Within contextual factors are both environmental (e.g., an accessible environment) and personal factors. As we discuss disparities in health and health care outcomes experienced by persons with communication disabilities, it is important to recognize that the complex factors contributing to the disparities are likely the result of both individual and contextual factors.

## Health and Health Care Disparities Experienced by Persons With Communication Disabilities

As previously mentioned, in the fields of public health and medicine, disability is often described or assumed to be an unhealthy state. This begins with the belief that an illness or traumatic event or accident is unhealthy. Since illness or a traumatic event can then lead to a disability, then an unhealthy state is equated with disability by association. Efforts are directed at preventing the illness or accident, thereby avoiding disability. When prevention fails, disability occurs. Any poor health outcomes, such as diabetes, cancer, arthritis, and so forth, are then directly related to the disability. Again, in the medical model, the solution to mitigating poor health outcomes is to intervene at the individual level. This is a simplistic view though, as highlighted by the contrasting ICF model.

I postulate that when disability interacts with a host of social, personal, and environmental factors, poor health and health care outcomes can result. Examples of these factors include poor social support, lack of accessible housing, decreased access to exercise opportunities, low socioeconomic status, decreased access to employment opportunities, and decreased access to high-quality health care. These factors have all been demonstrated in the literature to be more prevalent for individuals with disabilities and associated with poor health and health care outcomes (Iezzoni, 2011). There is a recent movement to describe these factors as social determinants of health (SDH). The Healthy People 2020 report, which is published by the Department of Health and Human Services and sets goals to improve the health and well-being of the population for the proceeding decade, described SDH as the economic and social conditions that influence the health of individuals (Office of Disease Prevention and Health Promotion, 2020). They establish five domains of SDH: neighborhood and built environment, health and health care, social and community context, education, and economic stability. To truly address the health needs of a population, it is necessary to consider and intervene on these more complex structures to make a significant and lasting impact. For example, if individuals live in a neighborhood with few grocery stores that sell fresh and affordable fruits and vegetables, the individuals are less likely to regularly eat these healthy foods, instead eating processed foods with higher sugar and fat content. Educating the individuals about healthy eating will not be an effective strategy to change their diet because healthy foods remain inaccessible. Instead, interventions are needed to increase access to healthy foods in the neighborhood.

The following is a further example from a study colleagues and I conducted in which we video-recorded clinical encounters of patients with aphasia (Morris et al., 2015). During one video-recorded primary care appointment, a physician noted that the patient, a middle-aged man who had a stroke several years prior and had right-side hemiparesis, had gained weight since the last appointment. The patient, with very limited speech abilities, affirmed the physician's question about not exercising regularly. The physician then replied with asking the patient if he was “lazy,” to which the patient shrugged. The physician immediately determined that the problem was rooted in the patient's behavior and, therefore, the solution was for the patient to change. Another approach could have been to ask the patient about his opportunities to exercise, including if the patient lived in a neighborhood in which he had a safe way to exercise given his physical and speech impairments (SDH domain: neighborhood and built environment). The physician also chided the patient for watching subjectively “too much” television. Again, a more nuanced approach would be to inquire about the patient's opportunities to participate in social or community activities that could be adapted for his physical and speech impairments (SDH domain: social and community activities).

The understanding and acceptance of the effects of SDH on underserved populations, such as those from racial and ethnic minority communities, continues to expand. In seeking to understand the complexity of SDH on health care and outcomes, many recommendations and efforts have been made to systematically collect patients' SDH within the medical setting (Gold et al., 2017; Gottlieb et al., 2014, 2015). Upon recognizing patients' SDH concerns, patients can then be offered connections to services as an integrative way to mitigate the effects of SDH. For example, a social worker available to serve in a primary care clinic could offer resources to patients with housing insecurity, such as connections to organizations that provide safe housing. Increased documentation of SDH and disability data in the electronic health record is an opportunity for these data to be intervened upon to better serve patients with disabilities. A large gap remains though in the documentation of patients' disability data in the electronic health record, which will be discussed later in this review article.

### Population of Persons With Communication Disabilities

To begin our discussion of health and health care disparities, it is necessary to define our population. I will focus on persons with speech, language, and voice disabilities, which I will define collectively as communication disabilities. Another article in this Special Forum will focus independently on hearing disabilities (see McKee et al., 2022). The most comprehensive data we have on communication disabilities and health and health care disparities come from the National Health Interview Survey (NHIS). The NHIS is a cross sectional population-level survey administered annually by the U.S. Census Bureau (U.S. Department of Health and Human Services, 2012). The survey is administered to community dwelling adults throughout the United States. In 2012, the NHIS included a speech, language, and voice supplement, sponsored by the National Institute on Deafness and Other Communication Disorders. Based on NHIS data, 10% of the noninstitutionalized U.S. adult population reported a speech, language, and/or voice disability (Morris et al., 2016). A higher percentage of people who identified as Black, American Indian/Native American, or “other race” reported a communication disability (10.2%, 11.6%, and 15.7%, respectively; Morris et al., 2016). The frequency of marriage among those who identified with a communication disability ranged from 36% for those with speech, language, and voice disabilities to 50% of those with voice only disabilities (compared to 54% of those without a communication disability; Stransky et al., 2018). Additionally, 36%–57% were employed (compared to 62% of those without a communication disability), and 13%–25% were below the federal poverty line (compared to 13% of those without a communication disability; Stransky et al., 2018).

### Patients With Communication Disabilities and Health Care Disparities

Health disparities are disparities in health outcomes, such as diabetes, cancer, and days sick. These disparities result from a wide range of medical, social, and environmental factors. Health care disparities are gaps in access to high-quality health care services and can lead to health disparities. According to the Phases of Health Care Disparities model in Kilbourne et al. (2006), there are three phases of health care disparities research. The first phase is “detecting” and includes defining the disparity, defining the underserved population, measuring the disparity in the underserved population, and considering the selection effects and confounding factors that affect the disparities (Kilbourne et al., 2006). The second phase is “understanding” and includes identifying determinants at the patient, clinical encounter, provider, and health care system levels. The third and final phase is “reducing,” which includes intervening, evaluating, translating, and dissemination, and changing policy that focuses on decreasing disparities.

#### Detecting Disability Health and Health Care Disparities

Across all types of disabilities, a growing body of evidence demonstrates that persons with disabilities, including communication disabilities, experience significant disparities in health outcomes (Iezzoni, 2011). Using findings from the aforementioned NHIS, people with communication disabilities have significantly higher rates of multiple chronic conditions (Stransky et al., 2018). These results differ by type of communication disability, with 40.4% of those with voice only and 62.7% of those with speech, language, and voice disability having two or more chronic conditions. In comparison, 24.5% of those without a communication disability have multiple chronic conditions, controlling for other demographic characteristics, such as race, ethnicity, age, and gender (Stransky et al., 2018). More specifically, persons with communication disabilities consistently have significantly higher rates of hypertension, cardiovascular disease, stroke, emphysema, asthma, cancer, diabetes, and arthritis (Stransky et al., 2018). Again, this is controlling for demographic characteristics, as well as the presence of other types of disabilities (e.g., physical, mental health, cognitive, and visual disabilities). Unfortunately, because the data collected are cross sectional, it is impossible to determine which occurred first, the chronic condition or the communication disability. Of course, some of the chronic conditions, such as a stroke, are strongly correlated with and can cause communication disabilities. Nonetheless, this cannot explain the high rates of all of the chronic conditions, such as arthritis, in people with communication disabilities. Additionally, regardless of which occurred first, chronic conditions require medical management and additional effort, such as regularly taking medications, regular medical visits, lifestyle changes, and so forth, all of which could be more challenging if a patient has difficulty communicating or communicates in alternative formats, such as those who use AAC or use American Sign Language.

In addition to disparities in health outcomes, persons with disabilities, including communication disabilities, experience disparities in the receipt of and access to high-quality health care (Iezzoni, 2011). For example, multiple studies report that women with physical or cognitive disabilities have significantly decreased rates of cancer screenings (e.g., 30% less likely to receive a mammogram and 40% less likely to receive a Pap test as compared to women without disabilities; Iezzoni et al., 2015, 2016a, 2016b). Specific to patients with communication disabilities, individuals with communication disabilities are more likely to experience a preventable adverse medical event in the hospital compared to those without communication disabilities (Bartlett et al., 2008; Sullivan & Harding, 2019). Additionally, in large surveys, patients with communication disabilities are more likely to rate the satisfaction with the quality of their care lower than those without a communication disability (Hoffman et al., 2005). Using the NHIS data, while patients with communication disabilities report high levels of having an established source of care to rely upon when they are ill or for routine care, they also report significant difficulties with finding a provider who will see them, with up to 10% of those with speech, language, and voice disabilities having difficulty (Stransky et al., 2018). Additionally, they reported significantly higher rates of delaying or foregoing medical care due to cost and availability barriers. Multiple qualitative studies involving individuals with a range of communication disabilities report challenges communicating with their health care teams (Balandin et al., 2007; Morris et al., 2013, 2014, 2021; Murphy, 2006; Nordehn et al., 2006). They report often feeling ignored, disregarded, and underestimated in health care settings. Because of this, many reported dissatisfaction with the quality of the communication with their provider and the care that they receive. It should be noted that all of the following health and health care disparities are likely compounded in individuals who live at the intersection of race/ethnicity and communication disabilities.

While the aforementioned studies have established a foundation for identifying health and health care disparities experienced by persons with communication disabilities, much more work needs to be done. The 2012 NHIS Supplement was an important first step. Unfortunately, this was a one-time supplement and provided only cross-sectional data. Section 4302 of the Patient Protection and Affordable Care Act mandated the inclusion of disability questions in all federally funded surveys (U.S. Department of Health and Human Services, 2011). While a significant advancement, historically, federal surveys—such as the American Community Survey (also known as the Census)—do not include communication disabilities in the list of disability questions (Morris & Hasnain-Wynia, 2014). To fully understand the health and health care outcomes of patients with communication disabilities, questions about communication abilities need to be consistently included in both cross-sectional and longitudinal population-based surveys. The Washington Group, which is an international group focused on disability statistics that was commissioned by the United National Statistical Communication, recommends the question: “In your usual language, do you have difficulty communicating, for example, understanding or being understood?” (Washington Group on Disability Statistics, n.d.).

#### Understanding Disability Health Care Disparities

The contributors to health and health care disparities for persons with communication disabilities are diverse and multifaceted. For the purpose of this review article, we will focus on health care disparities. In 2003, the Institute of Medicine (now National Academies of Medicine) released a report entitled *Unequal Treatment* (Institute of Medicine, 2003). The report outlined the evidence for racial and ethnic minority health care disparities. It provided a model stating that the factors contributing to differences in care between minority and nonminority patients fall within three categories: (a) clinical appropriateness and need, patient preferences, (b) operation of the health care systems and legal and regulatory climates, and (c) providers' bias, stereotyping, and various uncertainties when interacting with minority patients. While all three factors contribute to differences, the latter two contribute to disparities. While Unequal Treatment focused on racial and ethnic minority patients, similar patterns of disparities and causal factors have been observed in other patient populations, including those with communication disabilities. The following is a description of the patterns in persons with disabilities, including communication disabilities.

*Operation of the health care systems and legal and regulatory climates*. Inadequate research exists on the effects of the operation of the health care systems and legal and regulatory climate for persons with communication disabilities, yet there are some policy requirements. The Americans with Disabilities Act of 1990 requires health care organizations to provide “effective communication” to all patients with disabilities, including those with communication disabilities (U.S. Department of Justice, 2014). Despite this mandate existing for over 30 years, huge gaps remain to achieve effective communication in health systems. As evidence for this, the Department of Justice reports that complaints about the lack of effective communication are the most prevalent American With Disabilities Act lawsuit in the health care setting (United States Department of Justice Civil Rights Division, 2021). More recently, Section 1557 of the Patient Protection and Affordable Care Act requires that health care organizations “provide effective communication, which includes provision of auxiliary aids and services such as hearing assistive devices” (U.S. Government Publishing Office, 2016). Again, little evidence exists on how to provide these aids and services in the health care setting, particularly in the outpatient setting. In the inpatient setting, researchers have thoroughly studied the implementation of AAC aids and devices to address communication breakdowns for patients with temporary or long-term communication disabilities in the intensive care unit (Baxter et al., 2012; Happ et al., 2014; Heard et al., 2017; Holm et al., 2020). Findings from these inpatient-focused studies provide a potential foundation for how to implement communication aids and services in the outpatient setting.

*Providers' bias, stereotyping, and various uncertainties when interacting with minority patients*. Since the release of Unequal Treatment, researchers studying racial and ethnic disparities have explored the effects of health care providers' bias, beliefs, and uncertainty on health and health care outcomes, and have made progress identifying interventions that address and mitigate the effects of bias and beliefs. While we are further behind in the area of disability disparities, there has been foundational work to describe providers' biases, beliefs, and uncertainties. In a recent national survey of practicing physicians administered by Iezzoni et al. (2021), they found that the majority of the physicians had erroneous negative views of the lives of people with disabilities. Additionally, only 40.7% stated they were confident in providing the same quality of care to patients with disabilities, and just over half (56.5%) reported strongly welcoming patients with disabilities into their practice (Iezzoni et al., 2021). Numerous qualitative studies have found that persons with disability describe biases of health care providers (Iezzoni et al., 2004; McClintock et al., 2018; Mitra et al., 2016; Morris et al., 2013; Nicolaidis et al., 2015). These experiences cross cut disability types and health care environments. Women with mobility disability describe their clinicians' surprise and confusion when patients ask to discuss reproductive health, upending the erroneous assumption that women with disability are not sexually active (Iezzoni & Mitra, 2017). Patients across all types of communication disabilities—aphasia from a stroke, dysarthria from amyotrophic lateral sclerosis, aphonia due to a total laryngectomy, autism—report that their cognitive level is consistently questioned and they are often excluded from health care decisions (Burns et al., 2015; Morris et al., 2014; Nicolaidis et al., 2015). In a qualitative study we conducted with patients with diverse disability types, participants reported that increasing providers' knowledge and their recognition of bias was a top priority for improving the quality of care they received (Morris et al., 2021). Finally, multiple studies have found that health care professionals report feeling ill-prepared to care for and communicate with patients with disabilities, including communication disabilities (Ralston et al., 1996; Wilkinson et al., 2012; Zivianni et al., 2004). Research is needed to determine how to address bias, knowledge, and training of providers. Upon understanding the complexity of the factors that contribute to health care disparities, we can explore the third and final phase of health care disparities research.

#### Reducing Health Care Disparities Experienced by Persons With Communication Disabilities

The third and final stage of the model to address disparities in care is “reducing,” which is the stage in which we have the least amount of evidence. While descriptive research is still needed to elucidate the disparities and the factors that contribute to the disparities, there is a clear and urgent need for evidence-based interventions that address disparities in care for persons with communication disabilities. Unfortunately, we know that it can take years to develop such interventions and, even when developed, it can take decades for that evidence to become standard practice (Westfall et al., 2007). People with communication disabilities are experiencing adverse outcomes now and cannot wait decades until the descriptive work is completed and solutions implemented in clinical practice. Instead, innovative methods and study designs are needed in order to accelerate the research to practice pipeline. This will likely require significant collaboration between patients, researchers, policy makers, and health care organizations in order to efficiently develop, evaluate, and implement evidence-based interventions. The following is a case example that demonstrates this type of collaboration.

#### Case Example: Disability Equity Collaborative

Early in my research career, I became interested in determining a manner in which to identify persons with communication disabilities in the electronic health record (EHR), with the goal of tracking the quality of care delivered to the population at an organization level. I quickly realized there was not a straightforward manner to identify patients with communication disabilities in the EHR, and so proceeded to embark on a series of studies aimed at addressing the gap (Morris & Hasnain-Wynia, 2014; Morris & Kho, 2014; Morris, Lagu, et al., 2017; Morris, Schliep, et al., 2017; Morris et al., 2018, 2019). After several years, I began receiving phone calls from individuals working within health care organizations who were interested in documenting patients' disability status in the EHR. Many of the individuals were recently hired into their positions and were oftentimes the first person in their role. Often, the impetus for their position was the mandate of Section 1557, requiring health care organizations to designate an employee to oversee all disability accessibility initiatives (U.S. Government Publishing Office, 2016). Fundamental to any accessibility initiative, a health system needs to collect patients' disability status in order to provide accommodations to the appropriate patients. In the conversations about documenting disability status, many expressed uncertainties in their role and the initiatives they were implementing. Unfortunately, no evidence exists on how to best implement accessibility initiatives and the provision of accommodations within health care organizations. In response to the immediate need of these health systems, we developed a learning collaborative.

The learning collaborative model was first described by the Institute for Health Care Improvement (IHI) in 2003 (The Breakthrough Series: IHI's Collaborative Model for Achieving Breakthrough Improvement, Institute for Health Care Improvement, 2003). The goal of learning collaboratives is to bring together teams from hospitals and clinics to focus on improving care in a specific topic area. The innovators of learning collaboratives report that engaging organizations in “making real, system-level changes that would lead to dramatic improvements in care” is a method to integrate existing evidence with real-world experience, providing participants an opportunity to say to each other: “I had a similar problem. This is how I solved it.” Consequently, learning collaboratives build collaboration, offer abundant opportunities for shared learning, and create a system for organizations to support each other as they implement novel interventions. The IHI reports that they have sponsored dozens of learning collaboratives and have seen significant results, such as reducing ICU costs by 25% and reducing hospitalizations for patients with congestive heart failure by 50% (Institute for Health Care Improvement, 2003).

In 2018, we began our Learning Collaborative modestly with approximately five health systems involved. The goal of the group, which is named *Leaders*, was to provide an opportunity for members to share challenges, brainstorm solutions, and offer advice to each other. In 2019, we received funding from the Patient Centered Outcomes Research Institute (PCORI) through an engagement award to formalize the group, build resources, and establish an infrastructure to facilitate sustainability. Currently, over 50 health systems who represent over 250 hospitals and hundreds of outpatient clinics participate in biweekly virtual meetings and an active online community. For the virtual meetings, we regularly invite external experts to come and speak with the group. For example, speech-language pathologists have presented on communication aids to use with patients with limited communication in the inpatient setting. Additionally, external groups, such as policy makers and advocacy organizations, have requested the opportunity to speak with the group in order to solicit feedback from our members on their initiatives, programs, and materials in development. The group, which has only grown through word of mouth, was critically important to its members at the beginning of the COVID-19 pandemic, as health systems scrambled to implement masking and restricted visitor policies, while ensuring accessibility to patients with disabilities. As an example, the members collaboratively worked together to develop an exception policy for restricted visitor policies for patients with disabilities that they then implemented within their own organizations. These exception policies allowed for caregivers to be present in the hospital with a patient with communication disabilities to assist in communication and care for the patient. In 2021, Leaders received the American Speech-Language-Hearing Foundation Colorado State Clinical Achievement Award.

As a part of the Engagement Award from PCORI, we conducted interviews with 55 stakeholders including policy makers, researchers, advocates, researchers, professional societies, and payers. The goal of the interviews was to determine policy, practice, and research priorities for advancing disability equity in health care. Subsequently, we hosted a virtual summit to present the findings and collaboratively identify next steps. A total of 80 people representing a wide range of stakeholders participated in the summit. As an outcome of the Summit, we developed the Disability Equity Collaborative (DEC, https://www.disabilityequitycollaborative.org/). DEC aims to bring together stakeholders around key issues that need to be addressed in order to work toward disability equity in health care. As a part of DEC, we have multiple working groups including the aforementioned Leaders workgroup, a research workgroup, a guidelines and standards workgroup, and a documentation workgroup. The documentation workgroup is a prime example of what DEC aims to accomplish. The workgroup includes health care organizations, researchers, EHR vendors, payers, and patient advocates. Together, the group identified a specific research need within the documentation initiative: understanding health systems' current processes for documentation and clarifying common factors that are barriers to providing appropriate accommodations to their patients. Our team conducted additional interviews with health systems to explore this more deeply and reported the results to the group. The findings are being used by the EHR vendors as a starting point to develop a standardized build to offer to health systems that would allow the infrastructure to document patients' disability status in the EHR. Over the upcoming year, the EHR vendor will travel to the health systems represented in the group to learn firsthand the needs of the health care systems. The goal is that within the year, we will have standards and recommendations for EHR vendors and health care systems on how to best collect and document patients' disability status in the EHR. Consistent and accurate documentation of patients' disability status will be a critical step in developing future interventions aimed at improving the quality of care delivered to patients with communication disabilities. This is an example of how researchers, policy makers, patients, and health systems can work together to solve problems that contribute to disparities in care in a more timely and effective manner.

## Conclusions

Currently in our country and health care system, there is increased attention and recognition of the disparities experienced by underserved racial and ethnic minority populations. This attention and the subsequent efforts are long overdue and are critical to advancing health and health care equity. Unfortunately, persons with disabilities, including communication disabilities, are under recognized and often not acknowledged in conversations about disparities and equity. It is our role as researchers and clinicians who work and engage with persons with communication disabilities to be advocates and bring attention to the unjust disparities people with communication disabilities experience. This begins by recognizing the definition and models of disability that we may be using in our work, whether implicitly or explicitly. We need to recognize the complex and potentially problematic environments that persons with communication disabilities work, learn, live, and receive care within. Next, we need to improve our data, continuing to gather data on the health and health care outcomes of persons with communication disabilities. Finally, we need to think creatively on how we can infuse concepts of equity within the work that we are doing. This will often include engaging stakeholders, including persons with communication disabilities in the development, conduct, and dissemination of research. This could also involve investment in research designs and methods that facilitate implementation and dissemination of evidence into the real-world settings. Collaboratively, taking these steps together will bring us closer to ensuring equity in health and health care outcomes of persons living with communication disabilities.

## Supplementary Material

10.1044/2022_JSLHR-22-00057SMS1 Presentation Video Presentation Video
